# Disposition of improving quality of life in older adults: the case of Lithuania

**DOI:** 10.1007/s40520-023-02687-2

**Published:** 2024-02-07

**Authors:** Inga Iždonaitė-Medžiūnienė, Laura Preikšaitienė

**Affiliations:** SMK University of Applied Sciences, Liepų g. 83B, 92195 Klaipėda, Lithuania

**Keywords:** Quality of life, Older adults’ quality of life, Measures/actions to improve quality of life, Assessment of the quality of life

## Abstract

**Background:**

Quality of life is a phenomenon that recently required lots of concern, especially for older adults, since healthy aging and longevity have become the focus in life. Most research on the quality of life addresses certain issues of older people having special diseases, health problems, and disorders. Our research is based on holistic quality of life empowering multiple areas of life/domains of older adults without addressing their diseases or health disorders.

**Aim:**

Our research aims at evaluating the quality of life of the research participants (older people), addressing their problematic areas and suggestions for better quality of life.

**Methods:**

The conducted research implied mixed methods as quantitative survey and reflection based on interviews. We chose participants from the III age university and the ongoing project “Healthy Aging Program”.

**Results:**

The research results showed the lowest ratings for emotional and physical health and the highest rating for social health. Also, older adults tend to avoid specifying precise measures to improve their quality of life and demonstrate a more conservative stance regarding the implementation of more radical changes in improving water consumption, exercising, meal planning, and enhancing psychosocial well-being.

**Conclusions:**

The overall quality of life rating was lower than the average. Older adults are not sufficiently prepared or educated to make significant changes to develop healthier habits in their quality-of-life improvement, though, they demonstrate concern about their quality of life.

## Introduction

### The relevance of the study

The phenomenon of aging includes biological, psychological, and social changes that occur in the human body over time. It is a complex natural process that affects all aspects of human life, including physical appearance, physiological functioning, cognitive functions, and overall well-being. Although various interventions and strategies can influence the speed and quality of aging, this phenomenon cannot be stopped. A healthy lifestyle, such as balanced nutrition, regular physical activity, stress management, and social engagement, can contribute to healthier aging, improve overall well-being, and enhance the quality of life in later years.

Quality of life is a concept of broader context and subjective assessment that describes an individual’s personal, social, physical, and psychological experience of life. It reflects how people feel about their life conditions, how they assess their abilities, relationships with others, and their environment, as well as how they perceive daily events.

Special attention is also paid to the aging population. The World Health Organization divides the elderly into three categories: those aged 60 to 74, seniors aged 75 to 90, and long-lived individuals over 90 [1, 2]. According to the World Health Organization’s data [3], by 2030, 1 in 6 people in the world will be 60 years and older: the proportion of people aged 60 and over has increased from 1 billion in 2020 to 1.4 billion. By 2050, the number of people aged 60 and over in the world will double (2.1 billion). WHO predicts that the number of people aged 80 and over will triple between 2020 and 2050, reaching 426 million. WHO states that while some of the health differences in older age may be due to genetics, the majority are driven by the physical and social environment, including living conditions and communities, as well as personal characteristics such as gender, ethnicity, and socio-economic status. The environment in which people live as children or even as developing individuals, along with personal traits, has a long-term impact on their aging [3]. This underscores the relevance of research on the quality of life of the aging population, which is still lacking in abundance. Often, studies are focused on individual aspects of quality of life, such as cognitive functions, social environment, physical health, and assessments of psychosocial well-being, which are often associated with certain health conditions. Therefore, holistic quality of life studies that encompass multiple areas of life and activities are particularly relevant.

The study aims to evaluate the quality of life of the 3rd age university and “healthy aging” project participants (older people), identifying their problematic areas and suggestions for better quality of life.

## Theoretical overview

The World Health Organization explains that health encompasses physical, mental, and social well-being. In this regard, mental health is defined as a “state of well-being in which an individual realizes their abilities, can cope with the normal stresses of life, can work productively, and can make a contribution to their community” [4]. The authors confirm through their research that older people can experience not only the stress that is common to individuals of various ages but is more characteristic in older life when functional capacities gradually decline, physical health problems arise, and the quality of life deteriorates.

In other studies [5] quality of life is related to the concept of well-being, which is defined as an integrated approach to action aimed at maximizing an individual’s potential. This requires maintaining a balance and a purposeful continuation of activity in a particular environment. Thompson, Demiris et al. [5] describe the multidimensional model of quality of life as presented in previous studies, emphasizing that an individual should be examined in their everyday environment based on dimensions such as physical well-being/fitness, mental and cognitive health, social well-being, and spiritual well-being. Scientific literature describes various models of quality of life/well-being, which may not necessarily encompass the same areas.

Dyussenbayev [2], detailing the stages of older age, describes the behavior of individuals aged 61–73. The author emphasizes in his research that during this period, people were inclined to teach others, philosophize, and sometimes even overdo it because they had accumulated a wealth of knowledge and extensive life experience. Furthermore, the author claims that people aged 74–85 often become more isolated, contemplative, and critical or, less self-critical. They tend to acquire traits of obstinacy and self-assured introversion, replacing the high mental activity with the inertia of psychophysiological processes, which, according to the author, characterizes the age of the wise. Additionally, people older than 85 years are typically sentimental, and a depressive behavioral style, which is often characteristic of long-livers, becomes a mirror reflection of expressive childhood. Emotionalism transforms into sensitivity, meaning that they become emotional and easily vulnerable without necessarily showing it outwardly [2].

The explanations of the stages of older age provided by the author reveal clear connections with the main areas of quality of life, which have a significant impact on an individual’s quality of life in older age. These areas include physical health, financial status, social relationships and their development, spiritual well-being, and intellectual and emotional health. Therefore, all these areas are directly related to the quality of life of older people.

It is also important to note that older people, as one of the vulnerable groups, have been more affected by the problems caused by the pandemic [6], which have a long-term impact on the quality of life, especially for older individuals. The results of the study indicate that healthcare providers should pay special attention to the use of effective psychological interventions to reduce the problems experienced by older people.

During the study conducted by Thadathil, Jose, and Varghese [7], it was found that the average quality of life score was highest when assessing physical health and social relationships, and lowest when assessing psychological health. The research data indicated that profession, higher income, being in a relationship, and the absence of concurrent illnesses are factors that contribute to a better quality of life. The overall conclusion of the study was that the average quality of life score was lower than the average in all evaluated areas, with the psychosocial domain being particularly affected. Therefore, it is necessary to create more recreational opportunities for older people [7].

Furthermore, research data on the relationship between physical activity and quality of life for older adults showed that higher physical activity is associated with a better quality of life [8]. Other systematic studies and meta-analyses confirm that physically active older individuals experience a better quality of life and may even improve cognitive function [8, 9]. Additionally, Rugbeer et al. [10] and Fernandes Pucci et al. [11] also demonstrate in their studies that the quality of life for older adults who engage in regular exercise is better. Considering the benefits of physical activity, Alikhajeh et al. [8] suggest that traditional workouts and physical activities have certain limitations for older individuals due to psychological and physical challenges associated with the aging process, especially for those dealing with joint pain and mobility issues. The authors affirm that regular physical exercises should be performed to ensure minimal health risks. As an alternative method suitable for older individuals with psychological and physical difficulties, water exercises can also be considered, where water is used as a means of improving mental health and pain relief [8].

The conclusions of the study conducted by Gerino et al. [12] also highlight the importance of social relationships for older adults, as reducing feelings of loneliness is a preventive factor in the healing process. Strengthening mental health is achieved through increasing resilience and self-sufficiency and reducing the feeling of loneliness. The authors support the results of previous studies that a higher level of resilience contributes to improving the quality of life on physical and psychological levels while reducing anxiety and potential symptoms of depression. Furthermore, a study conducted by Russell et al. [13] demonstrates a connection between reduced physical and mental health, food insecurity, and poor dietary quality in older adults. Often, aging is associated with individually occurring dietary changes, either voluntarily or due to changing health conditions, and even due to changes in sensory acuity, which affect taste and smell perceptions, leading to dissatisfaction with food, which can become a significant health risk [14]. Nutrient depletion is a significant factor with a negative long-term impact on health. Other studies *citing* Amarantos et al. (2001) and Govindaraju et al. (2018), also show that better nutritional status and dietary quality in old age are important factors that influence health, longevity, and quality of life [15].

Netuveli and Blane [16] conducted a review of studies on the perception of quality of life in older adults and suggested that older age groups are unique and vulnerable due to declining physical and mental abilities, withdrawal from the job market with increased dependence on pensions, separation of family members, and/or isolation due to the death of peers, especially spouses or partners. After analyzing the studies conducted by other researchers (*citing* Xavier et al. 2003; Bowling et al. 2003; Gabriel et al. 2004; Wilhelmson et al. 2005), Netuveli and Blane [16] concluded that older adults rated their quality of life positively compared to others, placing a strong emphasis on social contacts, especially with family and children, health, financial status, and activities. However, negative assessments also emerged, highlighting their dependence on others due to functional limitations, feelings of unhappiness, and a decrease in social contact due to the loss of friends and/or family members [16]. These studies confirm that quality of life is not solely about health; for older adults, good social relationships, activity, and the ability to participate in meaningful activities without functional limitations and physical difficulties are crucial. Moreover, older individuals perceive their aging experience and its impact on quality of life with an understanding of aging, as they undergo significant age-related processes such as declining health and physical functioning [17]. Even a study conducted by Munawar et al. [18] (*citing* Phelan, Anderson, Lacroix, Larson, 2004) showed that more than 93% of older adults considered being able to remain independent from their surroundings to be a more important factor for quality of life than having good health.

The completed analysis of the scientific literature allows us to conclude that the perception of quality of life and active engagement in its improvement are highly individual and vary across different age groups. Since the concept of quality of life encompasses various life domains, older adults perceive aging processes and their impact on quality of life, particularly when faced with health issues and physical function decline. Conducting quality of life studies that assess various life domains for older adults could help identify key interventions through which older adults can enhance their quality of life through their engagement.

## Methods

The research conducted is of a mixed nature that included two studies: first quantitative, and second qualitative.

### Quantitative study

In the *quantitative study*, there were 252 participants from the III Age University and participants from the “Healthy Aging Program,” which included individuals aged 60 and above. The research sample was selected using a convenient sampling method, where researchers had actual access to the research participants through health and wellness projects and III Age University for this population.

In the quantitative study, six areas of quality of life were evaluated: physical, financial, social, intellectual, emotional, and spiritual health. The research instrument (questionnaire) based on literature analysis [16, 17, 24] consisted of a quantitative assessment of the quality-of-life areas. These areas were detailed with statements, which were evaluated on a 10-point scale, where 1 means completely disagree, 10 means completely agree, and other options are intermediate. Quality of life was measured using a scale with a score ranging from 0 to 10 points, where 0 meant the worst imaginable quality of life status and 10 the best. For this study, the quality-of-life score was arbitrarily divided into three categories: 0–4 points—poor quality of life, 5–7 point—saverage quality of life, 8–10—good quality of life. The quantitative data were processed by calculating averages.

### Qualitative study

In the qualitative study, we limited the sample to 20 research participants based on the principle of data saturation, which validates the choice of the research sample size (sufficiency). Data saturation in qualitative research refers to the point when new data cease to yield additional insights or information. The justification for our qualitative research sample was based on the practices of other researchers who conducted qualitative studies earlier. For instance, Hennink and Kaiser [19] analyzed 23 scientific articles that used empirical data (*n* = *17*) to assess data saturation and confirmed that in qualitative empirical research, data saturation occurs when we include between 9 and 17 research participants. Gugiu et al. [20] found that out of 15 concepts identified as most important in their qualitative research, 90% of them emerged with an *n* = *8* research sample, and 100% with an *n* = *14* sample. Additionally, previous qualitative studies on nursing work have suggested that data saturation is achieved after 12 interviews [21]. Sebele-Mpofu [22] explains that while some researchers argue that data saturation is achieved through 12–30 interviews, very few of them specify the sample size or provide a rationale for its selection.

In our qualitative study (reflexive), research participants were selected from those who participated in the quantitative study. Moreover, Mandal [23] emphasizes that there is no clear indicator to definitively determine when data saturation has occurred. According to the author, researchers often have to limit their studies based on practical constraints, as resources may become limited. Thus, the quality of a study is often influenced by factors such as time, energy, and finances, rather than solely by the adequacy and objectivity of the research sample [23], *citing* Green & Thorogood 2004. Thus, this brief literature summary confirms the reliability of the chosen research sample.

In the qualitative study, the research participants reflected on their assessment of the quality of life and revealed their intentions or disposition to improve the quality of life based on standardized open-ended questions (interviews). When reflecting, the research participants had to identify which areas were problematic, anticipate possible ways/tools to solve these problems and express personal commitment to implementing these solutions themselves. The qualitative content analysis was used to present key aspects of the research participants’ reflections during the interviews.

### Measurements

Data collection and instruments for the research. In the quantitative study, six areas of quality of life were evaluated: physical, financial, social, intellectual, emotional, and spiritual health. The research instrument (questionnaire) based on literature analysis [16, 17, 24] consisted of a quantitative assessment of the quality-of-life areas. These areas were detailed with statements, which were evaluated on a 10-point scale, where 1 means completely disagree, 10 means completely agree, and other options are intermediate. Quality of life was measured using a scale with a score ranging from 0 to 10 points, where 0 meant the worst imaginable quality of life status and 10 the best. For this study, the quality-of-life score was arbitrarily divided into three categories: 0–4 points—poor quality of life, 5–7 points—average quality of life, 8–10—good quality of life. The quantitative data were processed by calculating averages.

In the qualitative study, the research participants reflected on their assessment of the quality of life and revealed their intentions or disposition to improve the quality of life based on standardized open-ended questions (interviews). When reflecting, the research participants had to identify which areas were problematic, anticipate possible ways/tools to solve these problems and express personal commitment to implementing these solutions themselves. The qualitative content analysis was used to present key aspects of the research participants’ reflections during the interviews.

### Research ethics

Research ethics were ensured by implementing the following principles of research ethics:

1. *Principle of anonymity*—the research participants did not disclose any personal information that could help identify a specific individual.
2. *Principle of confidentiality*—the researchers committed not to disseminate important information (research data and results) without the consent of the research participants.
3. *Principle of fairness*—the research participants committed to providing accurate and truthful data/evaluations, while the researchers committed to providing accurate data analysis and results and informing research participants accordingly.
4. *Principle of transparency*—the research participants were informed about the research being conducted, its purpose, objectives, benefits, and results. The research participants were informed that they had the opportunity to withdraw from the study at any stage.
5. *Principle of voluntariness*—the research participants were allowed to independently and freely decide whether to consent to participate in the study.

## Research results

To assess the quality of life of the research participants (elderly individuals), identify problematic areas, and anticipate possible solutions, a mixed-methods study was conducted. The quantitative part of the study allowed research participants to assess their quality of life in six domains on a scale of 1 to 10 based on pre-established statements. The average ratings of research participants are presented in Table 1.

**Table 1:** Quality of life assessment by areas (quantitative research results, 2023)

Quality of life areas	Average ratings (10-point scale)	Quality of life level*
Physical health	4.9	Poor
Financial health	5.22	Average
Intellectual health	5.29	Average
Emotional health	4.81	Poor
Social health	5.31	Average
Spiritual health	5.28	Average
The overall quality of life	5.16	Average

*0–4 points–poor quality of life, 5–7 points–average quality of life, 8–10–good quality of life

The research data indicate that the research participants (older age) rate their quality of life at 51.6% of the maximum score (100%). All six areas were rated similarly and did not exceed 55%. The lowest ratings were given to emotional health (48.1%) and physical health (49%). The research participants rated the social health area as the highest (53.1%), although this rating was not very high in general as well.

Another part of the research, which is qualitative, focused on the reflection of the research participants (older age) regarding the quality-of-life areas assessed in the first part of the research. The reflection highlighted the following key aspects: the main measures that the research participants could take to improve their quality of life, the initial steps that the research participants intended to take, and the elements of quality-of-life control that the research participants could apply themselves. When reading the reflections of the research participants, the main measures that could be implemented to improve the quality of life of the older age research participants were identified (Table 2).

**Table 2:** Key measures to improve the quality of life of older age individuals. Source: Compiled by the authors based on the research results, 2023

Types of key measures	Key measures (indicated by research participants)	Benefit to research participants, clarity of implementation
Passive intentions	To focus on nutrition/eating	The benefit is unclear, implementation is unlikely, and specific actions are not anticipated or specified
	To focus on psychology	
	To manage emotions	
	To focus on active movement for 30 min	
Active intentions	To engage in sports	The benefit is possible in cases where the activity is implemented, still specific actions, duration, and other details are not anticipated or specified
	To control/monitor eating	
	To increase water intake per day	
	To get some medicine	The benefit is experienced when taking medications
Longer term intentions	To participate in similar projects	The benefit is questionable, and there is uncertainty regarding another similar project

The measures identified by the research participants to improve their quality of life were categorized into 3 types: active intentions, passive intentions, and long-term intentions. The research data show that the identified measures by the research participants were more intentions rather than concrete actions. The passive intentions involve focusing on nutrition and psychology without clearly indicating the specific actions to be taken, as focusing attention is not a concrete active step. This raises questions about what exactly needs to be done and what results are expected. Similarly, emotional management is a rather broad concept description, which does not necessarily mean that individuals fully understand its essence and likely cannot specify the activities that will be undertaken to acquire the ability to manage emotions or how it would be practiced. Another measure attributed to passive intentions is “to focus for 30 min on active activity,” as the mere focus does not ensure the actual performance of physical activity.

While analyzing active intentions, it is positive that the research participants intended to monitor their eating habits—when, what, and how they wanted to eat. However, this does not guarantee clear changes in eating habits, as the introduction of changes in eating habits into the daily routine was not specified. Additionally, the intention to exercise or increase daily water consumption also remains unspecified and loosely related to the acquisition of these habits. The least likely implementation of changes was expected for those participants who planned to participate in similar projects in the future without specifying concrete actions to improve their quality of life. It is evident that the research participants (older adults) tend to avoid specifying precise measures to improve their quality of life, and they are insufficiently prepared or educated to change their habits or commit to change.

After identifying the main quality of life measures, the first steps that the research participants intend to take were highlighted (Table 3).

**Table 3:** The first steps the research participants intend to take to improve their quality of life. Source: Compiled by the authors based on the research results, 2023

Action types	Actions (indicated by research participants)	Explanation of activity implementation
Eating control	Reduce sweets, snacks, late-night eating	Unplanned snacking on sweets, late-night eating scale—frequency, quantities, and what will be reduced
	Check on what and how much I eat during the day	The purpose of daily meal marking is unclear if there are no planned changes
Improvement of psycho-emotional state	Dedicate time to psychology	The allocation of time for psychology is unclear—specific actions (e.g., reading psychological literature, self-analysis, learning, etc.) are not detailed
	Interact with positive people	Undetailed communication with positive people—how they will appear, who they are and where they are, how to interact with individuals lacking positivity, etc
	Undergo psychotherapy	The execution of psychotherapy is not explained—where, when, and how it will be done, whether a psychotherapist will be involved, etc
Medication-based health improvement	Meet with the Doctor get medications	A specific specialist is explicitly mentioned, the scheduled appointment and the purpose of the visit are specified, and the purchase of medications is presented as an inevitability
Improvement of physical and functional condition	Engage in physical activity, exercise	The execution of physical activity is not detailed—there is no clarity on where, when, how, how consistently, and what will be performed
Learning to improve the quality of life	Use accumulated knowledge	A general reflection is presented that accumulated knowledge should be applied
	Allocate time for reflection	There is an anticipated concern about the quality of life and an expressed need for reflections, but it is not detailed how or when the reflection will take place

The research data revealed that it was unusual for the research participants to specify their first actions, and what they intended to do to improve their quality of life. Anticipating these actions was a significant responsibility for the older research participants, which they were not inclined to take on immediately. The older research participants required a longer time frame to make decisions about changes, and some were not willing to drastically change their established habits, which were important factors influencing their quality of life. The research data indicated that both control of eating and improvements in psycho-emotional state, physical, and functional conditions were more theoretical statements than concrete actions planned for the implementation. The research participants did not foresee specific changes in eating habits, physical activity, and psycho-emotional state—they focused on only general intentions that may not necessarily become a reality. It was not enough to desire to reduce the consumption of sweets, snacks, late-night eating quantities, and frequencies, and not have a clear goal. It was insufficient to dedicate time to psychology without knowing what specific actions should be taken without a clarified understanding of the concept of “psychology.” The desire to engage in physical activity without a clear understanding of what activity to undertake, when, and how to do it was also insufficient. On the other hand, it was very positive that the research participants were willing to use accumulated knowledge and reflect on their actions, experiences, and the results of their changes. However, the lack of specificity limited the opportunities to apply the accumulated knowledge to improve the quality of life and to learn from reflecting on experiences and sensations.

During the research, when evaluating the quality of life of the research participants, attention was given to the frequency of self-control among the research participants to ensure that new habits and intended actions would bring about real changes and improve their quality of life. The research participants emphasized the following self-control frequencies: short-term control (daily, every 10 days, every 2 weeks), medium-term control (after 3 months), and long-term control (after 1 year, participating in another similar project). When starting to implement changes aimed at improving the quality of life, it was necessary to ensure short-term self-control first to establish new positive habits, and later the frequency of self-control can be less frequent.

In summary, the quality of life of the research participants (older adults) is evaluated very cautiously, with all the assessed areas of quality of life being rated similarly and not reaching 55 percent. The research participants exhibited a more conservative stance regarding the implementation of more radical changes in improving water consumption, exercising, meal planning, and enhancing psychosocial well-being. The research participants were not yet inclined to commit to more significant changes to develop new habits for a better quality of life.

## Discussion

In this study, we found that older people accept their quality of life as impacted by changes in various life domains. They evaluate their quality of life as average and perceive it as naturally changing. Previous research also reveals that older adults accept changes in various life domains that affect their quality of life [17]. Lifestyle changes focusing on balanced nutrition, regular physical activity, stress management, and social engagement contribute to healthier aging, improve overall well-being, and enhance the quality of life in later years [7, 8, 16].

This study data indicate that the quality of life of older adults is rated below average which compliments the previous research [16] stating that dependency on others due to functional limitations, a sense of disability, and reduced social contacts. Moreover, most elderly people consider the ability to be independent from their surroundings as a more important factor for quality of life than having good health [18]. The completed research data reveal that when outlining the key actions to improve quality of life, general statements dominate, they do not ensure clear opportunities for change. This witness that older adults have a lack of knowledge or simply are not self-motivated enough to actively engage in personal quality of life enhancement.

Improving the quality of life is one of the most important outcomes of elderly care. International-level healthy aging action plans affirm the importance of improving the quality of life, and interest in measuring the quality of life of older adults is growing internationally [25].

### Limitations of the study and future research

The study was conducted within a community of the III age university and the “Healthy Aging Program” project, potentially limiting the generalizability of the findings. The research sample included relatively healthy older adults, various illnesses and health problem issues were not evaluated and taken into consideration. In addition, the study is limited by the selected areas of quality of life (physical, financial, social, intellectual, emotional, and spiritual health) and the chosen sample size (252 participants in the quantitative study and 20 participants in the qualitative study), consisting of individuals aged 60 and older as well as geographical indicator (Lithuanian case). The study involved a relatively small number of participants, therefore, it may have limited results, so precaution should be taken into consideration when generalizing the research findings beyond the scope of their application. To conduct more detailed research on quality-of-life improvement in older age and to enhance the clarity of quality-of-life assessment results, further research is necessary by expanding the study’s sample size and including demographic variables of research participants (such as gender, education, place of residence, marital status, etc.), other geographical regions. More comprehensive studies on quality of life in older age will likely not only benefit the research participants but also contribute to the improvement of quality of life for other elderly individuals and further quality of life studies’ development.

## Conclusions

The quality of life of the research participants is about the average. It is assumed that older individuals are not sufficiently prepared or educated to make significant changes to develop healthier habits for their quality-of-life improvement. When outlining the key actions to improve quality of life, general statements dominate, and they do not ensure clear opportunities for change. The research participants either are not inclined to commit to changes or lack sufficient knowledge, skills, and understanding of how to enhance their quality of life. However, they demonstrated concern about their quality of life.

The study showed that the research participants think of planning quality of life changes and new habits with controlled frequency, choosing between short, medium, or long-term intervals. For those opting for medium and long-term frequency, it is recommended to start with short-term self-control actions to establish a positive habit of implementing changes and improving their quality of life.

Educating older adults about the possibilities and self-motivation to improve their personal quality of life is highly recommended.
